# Response of soil biological properties and bacterial diversity to different levels of nitrogen application in sugarcane fields

**DOI:** 10.1186/s13568-021-01331-4

**Published:** 2021-12-17

**Authors:** Shangdong Yang, Jian Xiao, Tian Liang, Weizhong He, Hongwei Tan

**Affiliations:** 1 Agricultural College, Guangxi Key Laboratory of Subtropical Bio-Resource Conservation and Utilization, Guangxi University, Nanning, 530004 China; 2 Guangxi Key Laboratory of Sugarcane Genetic Improvement, Guangxi Academy of Agricultural Sciences, Nanning, 530007 P. R. China

**Keywords:** Sugarcane, Nitrogen stress, Soil enzyme, Microbial biomass, Bacteria, Diversity

## Abstract

To select an eco-friendly nitrogen (N) application level for sugarcane production, soil fertility and soil bacterial diversity under different nitrogen application levels were analyzed. Four levels of urea applications were high Nitrogen (H, 964 kg ha^−1^), medium Nitrogen (M, 482 kg ha^−1^), low Nitrogen (L, 96 kg ha^−1^) and no Nitrogen (CK, 0 kg ha^−1^) treatments, respectively. The results showed that the soil microbial biomass carbon and phosphorus were altered significantly by CK and L treatments. Moreover, the indexes of soil bacterial richness and diversity in the sugarcane field could be significantly improved by L. At the genus level, *SC-I-84*, *Mycobacterium*, *Micropepsaceae*, *Saccharimonadales*, *Subgroup_2* and *Acetobacteraceae* were the unique dominant bacteria in the soil with the H treatment. *JG30-KF-CM45* and *Jatrophihabitans* were the unique dominant genera in the M treatment. *Subgroup_6*, *HSB_OF53-F07*, *Streptomyces*, *67–14*, *SBR1031* and *KD4-96* were the unique dominant genera in the L treatment. In contrast, *FCPS473*, *Actinospica*, *1921–2*, *Sinomonas*, and *Ktedonobacteraceae* were the unique dominant genera in the CK treatment. The findings suggest that soil fertility all could be changed by different N application levels, but the most increasing integral effect only could be found in L. Moreover, even though soil bacterial richness could be significantly promoted by the M and H treatments, but soil bacterial diversity could not be significantly improved. On the contrary, soil bacterial diversity and richness all could be improved by L treatment. In addition, higher abundance of unique soil dominant bacteria could be only found in L treatment which compared to the CK, M and H treatments. These findings suggest that the rate of 96 kg ha^−1^ N application is ecofriendly for sugarcane production in Guangxi.

**Supplementary Information:**

The online version contains supplementary material available at 10.1186/s13568-021-01331-4.

## Introduction

Sugarcane (*Saccharum officinarum* L.), an important sugar crop, is utilized as a source of biofuel and renewable bioenergy around the world (Chandel et al. 2011). China is the third-largest sugar-producing country in the world followed by Brazil and India (Raza et al. 2019). In China, approximately 90% of the sugarcane crops are planted in southern and southwest regions, including Guangxi, Guangdong, and Yunnan provinces (Yang et al. 2021). In particularly, Guangxi Province is the top sugarcane and sugar producer of China, accounting for more than 65% of the sugar production of the nation since 1993 (Li 2004). However, low sugarcane yield is still a major problem in China (Tayyab et al. 2021). To improve cane yield, chemical fertilizers were overused by farmers in Guangxi, China. The nitrogen (N) fertilizer is applied at 600–800 kg ha^−1^ annually for sugarcane in China, but only 60 kg ha^−1^ for newly planted canes and 80–120 kg ha^−1^ for ratoon canes are applied in Brazil (Li and Yang 2015). Moreover, overuse of chemical fertilizer not only negatively influences microbial systems but also disrupts terrestrial and aquatic ecosystem functions (Robertson and Vitousek 2009). A method to reduce chemical fertilizer inputs and enhance crop production in an ecofriendly manner is an urgent need for sugarcane production, particularly in Guangxi, China.

Nitrogen (N) is one of the important essential nutrients affecting the growth of crops and is also a key pillar of global food security (Mueller et al. 2012). N is a limited resource; even so, the yield and quality of crops are largely determined by plant demand for N (Hawkesford 2014). However, the N surplus, can cause massive losses through denitrification, leaching, volatilization, and runoff, making the soil unable to meet human demands for clean water, clean air, and abundant healthy food (Matson et al. 1998; Erisman et al. 2013). In contrast, N input consistently below crop N requirements (*i.e*., N lacks) leads to soil nitrogen extraction and soil quality degradation (Sanchez 2002; Sanchez and Swaminathan 2005). Currently, face a challenge in finding an effective balance between N input and crop N requirements to achieve high crop yields while maintaining soil quality and reducing the environmental footprint (Lassaletta et al. 2014; Zhang et al. 2015). Niu et al. (2021) found that water soluble fertilizer affected the enrichment of microorganisms by improving the nutrient content of the soil, thereby affecting the growth and yield of sugarcane. Meanwhile, Compton et al. (2004) also found that soil microbial biomass decreased with the application of nitrogen. And Yao et al. (2014) found that soil microbial diversity decreased with the increasing N deposition.

Soil quality depends on numerous physical, chemical, biological, biochemical and microbiological parameters (Chaer et al. 2009). In particular, the latter two parameters are the most sensitive indicators that respond rapidly to changes in soil quality (Bastida et al. 2008). Soil enzyme activity is not only a sensitive biochemical indicator of quality (Raiesi and Beheshti 2014) but is also capable of reflecting ecosystem processes (Doran and Zeiss 2000). However, enzymatic activity is presented only in absolute terms, and soil microbial biomass carbon (MBC), microbial biomass N (MBN) and microbial biomass phosphorus (MBP) are also used as tools for monitoring soil quality (Pandey et al. 2014). In addition, soil microorganisms play an important role in soil biogeochemical processes such as N, phosphorus and other element cycles (Urbanová et al. 2015). Soil microbial community composition and diversity are imperative to maintain soil health and crop productivity (Mangan et al. 2010).

Previous studies have also shown that soil microbial community structure and activity of soil microorganisms can be altered by fertilization (Guo et al. 2018; Chen et al. 2019). For example, the application of chemical N fertilizers is related to change in the community richness and the structure of ammonia-oxidizing bacteria (Chen et al. 2014). Meanwhile, excessive fertilization will ultimately affect the richness of nitrifying bacteria in the soil (Geisseler and Scow 2014). Therefore, in this study, we objectify to (1) compare soil fertility and (2) analyze the response of the soil bacterial community structure to different nitrogen applications.

## Methods

### Field site description and experimental designs

The samples were collected on May 12, 2020, from the Experimental Base of Sugarcane Research Institute, Guangxi Academy of Agricultural Sciences, where is located on Longan County (107°75′E and 23°17′N), Guangxi Zhuang Autonomous Region, China. The sugarcane variety Guitang49 was planted in early March 2019.

Four N treatments were applied as follows: application of 964 kg ha^−1^ urea with pure N input of 450 kg (H); application of 482 kg ha^−1^ urea with pure N input of 225 kg (M); and application of 96 kg ha^−1^ urea with pure N input of 45 kg (L) and no urea application (CK). And 300 kg ha^−1^ calcium, magnesium, and phosphorus were likewise applied in 4 different treatments as basal fertilizer. The conventional field management measures were carried out identically except for the differences in nitrogen application levels. Each nitrogen application pattern was randomly treated with three replications. A total of 12 plots, each plot area was 42 m^2^. In every plot, there were five rows, and the row length and space were 7 m and 1.2 m, respectively. The planting density was approximately 90,000 buds per hectare.

### Soil sampling and soil biological properties analysis

Soil samples were collected in May 2020 from 12 plots that represented all the treatments in different nitrogen application experiments. To collect soil samples, the auger was sprayed with 75% ethanol for disinfection firstly, and then soil samples were collected by sterilized auger with the same depth of 40 cm in each treatment plot. From each plot, soil samples were collected from 12 random sites and mixed well. These soil samples were collected in sterile plastic bags and placed on ice in an ice box. The samples were immediately transferred to the laboratory, where they were sieved through a 2-mm mesh stainless steel sieve, and then stored in a refrigerator at 4 °C for immediate analysis or were stored at − 80 °C for later use. Meanwhile, portions of the soil samples were air dried for soil chemical analyses (Yang et al. 2021). The chemical properties of the soil were as follows: pH 5.1, and the contents of organic matter, total nitrogen, phosphorus and potassium were 17.6 g kg^−1^, 0.92 g kg^−1^, 0.92 g kg^−1^ and 0.56 g kg^−1^, respectively. The contents of available nitrogen, phosphorus and potassium were 85 mg kg^−1^, 35.3 mg kg^−1^ and 125 mg kg^−1^, respectively.

### Soil enzyme activities

Soil microbial biomass carbon, nitrogen and phosphorus and the activity of enzymes such as *β*-glucosidase, phosphatase and protease were analyzed using the following methods:

*β*-Glucosidase (EC.3.2.1.21) assay was based on *ρ*-nitrophenol (*ρ*NP) release after cleavage of a synthetic substrate (*ρ*-nitrophenyl-*β*-D-glucoside). The color of released *ρ*-nitrophenol was measured at 400 nm in a spectrophotometer (UV-1700, Shimadzu, Japan). A standard curve was plotted using 0–80 μg mL^−1^ *ρ*-nitrophenol. The enzyme activities were expressed as *n* moles *ρ*NP released per g dry soil per minute (*n* mol *ρ*NP g^−1^ min^−1^) (Deng and Tabatabai 1994).

Acid Phosphatase (phosphodiesterase) activity in soils was estimated by measuring the amount of *ρ*-nitrophenol released after incubating the samples with *ρ*-nitrophenyl phosphate (Alef et al. 1995). In a reaction tube, 0.25 mL toluene, 4.0 mL modified universal buffer (pH 6.0; made by dissolving 12.1 g tris, 11.6 g maleic acid, 14.0 g citric acid and 6.3 g boric acid in 500 mL 1 M NaOH and making the volume 1 L) and 1.0 mL *ρ*-nitrophenyl phosphate (15 mmol L^−1^) were added to 1.0 g soil sample and the mixture was incubated at 37 °C for 1 h. The reaction was terminated by adding 1.0 mL of 0.5 mol L^−1^ CaCl_2_ and 4.0 mL of 0.5 mol L^−1^ NaOH to the mixture prior to filtration. The absorbance of released *ρ*NP was measured at 400 nm in a spectrophotometer (UV-1700, Shimadzu, Japan), and the phosphatase activity was expressed in mg *ρ*-NP g^−1^ h^−1^.

Aminopeptidase activity was measured by the method described by Pansombat et al. (1997) using 0.002 M N-benzoyl-Lxycarbonyl glycyl L-phenylalanine (ZGP). The absorbance was measured in a spectrophotometer at 570 nm wavelength. All the analyses were conducted in 5 replicates.

### Soil microbial biomass

Soil microbial biomass N (MBN) and soil microbial biomass C (MBC) were determined using the chloroform fumigation-extraction method as described by Brookes et al. (1985) and Vance et al. (1987). Soil microbial biomass P (MBP) was determined by the phosphorus molybdenum blue colorimetric method (Powlson et al. 1987).

### Analysis of soil microbial diversity

Microbial community genomic DNA was extracted from samples using the E.Z.N.A.® soil DNA Kit (Omega Biotek, Norcross, GA, U.S.) according to manufacturer′s instructions. The DNA extract was checked on a 1% agarose gel, and DNA concentration and purity were determined with a NanoDrop 2000 UV–vis spectrophotometer (Thermo Scientific, Wilmington, USA). PCR amplification and sequencing of total DNA extraction from rhizosphere soil samples were performed by Shanghai Majorbio Biopharm Technology Co., Ltd. The V3-V4 hypervariable region of the bacterial 16S rRNA gene was amplified with bacterial primer pairs 338F (5′-ACTCCTACGGGAGGCAGCAG-3′) and 806R (5′-GGACTACHVGGGTWTCTAAT-3′) by an ABI GeneAmp® 9700 PCR thermocycler (ABI, CA, USA). PCR amplification was performed by ABI GeneAmp® 9700 PCR thermocycler (ABI, CA, USA), and the PCR products were recovered by 2% agar-gel electrophoresis. The products were purified by an AxyPrep DNA Gel Extraction Kit (Axygen, USA) and quantified by a Quantus Fluorometer (Promega, USA). Purified amplicons were pooled in equimolar amounts and paired-end sequenced (2 × 300) on an Illumina MiSeq platform (Illumina, San Diego, USA) according to the standard protocols by Majorbio Bio-Pharm Technology Co., Ltd. (Shanghai, China). Raw reads were deposited in the NCBI Sequence Read Archive (SRA) database (Accession Number: SRP302628).

### Statistical analyses

Quantitative insights into microbial ecology (QIIME) (version 1.17) was used to truncate the 250 bp reads (average quality score < 20 over a 50 bp sliding window). Ambiguous reads were removed, followed by assembling of overlapped sequences containing longer than 10 bp sizes (Tayyab et al. 2021). Operational taxonomic units (OTUs) with 97% similarity cutoff were clustered using UPARSE (version 7.1, http://drive5.com/uparse/), and chimeric sequences were identified and removed. The taxonomy of each OTU representative sequence was analyzed by RDP Classifier (http://rdp.cme.msu.edu/) against the 16S rRNA database using confidence threshold of 0.7.

Statistical analyses were carried out by SPSS software using a multiple range test at a 0.95 level of probability to determine significant differences (*p* < 0.05) between the treatments. The results are shown as the standard deviation of the mean (mean ± SD). The experimental data were analyzed using Excel 2019 and Statistical Product and Service Solutions (SPSS) Statistics 21, and online data analysis was conducted by using the free online platform of the Majorbio Cloud Platform (www.majorbio.com) of Majorbio Bio-Pharm Technology Co., Ltd. (Shanghai, China).

## Results

### Soil enzyme activities

The trends in soil enzyme activity under different N applications are shown in Table 1. The activities of *β*-glucosidase and acid phosphatase in soil under high N application (H) were all significantly higher than the activities in CK. However, the activity of *β*-glucosidase in the M or L treatments was not significantly different between CKs. The activity of acid phosphatase in the L treatment was significantly higher than that of CK, but there was no significant difference between the M treatment and the CK. In addition, the activities of aminopeptidase in N applications were all significantly lower than those in CK, and there were no significant differences between each of the N applications. This result suggested that the activities of soil enzymes related to carbon, N and phosphorus cycles in soil were all affected by N application. The activities of *β*-glucosidase and acid phosphatase were significantly increased by higher N application, but the activity of aminopeptidase was significantly decreased by N application.

**Table 1:** Soil enzyme activities under four different N application treatments (nmol g^−1^ min^−1^)

Treatments	*β*-Glucosidase	Aminopeptidase	Acid phosphatase
H	0.25 ± 0.08a	14.00 ± 1.57b	0.42 ± 0.14a
M	0.17 ± 0.02ab	14.09 ± 0.20b	0.25 ± 0.06b
L	0.06 ± 0.01c	11.68 ± 1.87b	0.43 ± 0.04a
CK	0.12 ± 0.01bc	18.55 ± 1.22a	0.12 ± 0.03b

All data are presented as the mean ± SD (standard deviation)

*H* high N application in the sugarcane soil (964 kg ha^−1^), *M* medium N application in the sugarcane soil (482 kg ha^−1^), *L* low N application in the sugarcane soil (96 kg ha^−1^), *CK* no N application in the sugarcane soil (0 kg ha^−1^)

Different letters in the same column indicate significant differences between treatments at *P* < 0.05 among the means of the four treatments

### Soil microbial biomass

The soil microbial biomass N (MBN) in the different nitrogen application treatments was significantly higher than the MBN in the CK treatment. In contrast, soil microbial biomass carbon (MBC), except in the low N application treatment, was significantly lower than that in the CK. The soil microbial biomass phosphorus trend was similar with MBC except in the M treatment, which was not significant between the M treatment and CK. These results indicated that soil microbial biomass C, N and P were also significantly affected by N application. However, the trends of soil microbial biomass were dependent and affected by N application (Table 2).

**Table 2:** Effect of different N applications on soil microbial biomass C, N and P in sugarcane fields (mg kg^−1^)

Treatments	Microbial biomass C	Microbial biomass N	Microbial biomass P
H	112.42 ± 3.83c	29.23 ± 2.34b	44.50 ± 1.05c
M	12.91 ± 1.00d	35.85 ± 0.62a	202.41 ± 7.78b
L	146.65 ± 5.33a	20.20 ± 0.62c	242.16 ± 19.81a
CK	130.33 ± 9.26b	8.50 ± 0.81d	198.81 ± 8.97b

All data are presented as the mean ± SD (standard deviation)

*H* high N application in the sugarcane soil (964 kg ha^−1^), *M* medium N application in the sugarcane soil (482 kg ha^−1^), *L* low N application in the sugarcane soil (96 kg ha^−1^), *CK* no N application in the sugarcane soil (0 kg ha^−1^)

Different letters in the same column indicate significant differences between treatments at *P* < 0.05 among the means of the four treatments

### Soil bacterial diversity and richness

In Table 3, the Shannon index was significantly higher only in the low nitrogen application treatment than in the other treatments. The Simpson index showed the opposite trend to the Shannon index, which was significantly lower than those of the other treatments. In addition, the Ace and Chao1 indexes, which were used as indicators of bacterial richness, in the N application treatments were all significantly higher than those in the CK. Moreover, the highest Ace and Chao1 indexes were all shown in the low N application treatment, which was significantly higher than all the other treatments. This result suggested that soil bacterial diversity and richness in sugarcane fields could all be improved by N application. In particular, the greatest effect was shown by the low N application.

**Table 3:** Indexes of soil bacterial diversity and richness in sugarcane fields under four N application treatments

Treatments	Shannon index	Simpson index	Ace index	Chao1 index	Coverage
H	6.19 ± 0.06b	0.0053 ± 0.0001a	2726.32 ± 276.81b	2697.06 ± 257.66b	0.98
M	6.19 ± 0.15b	0.0052 ± 0.0011a	2902.96 ± 279.16b	2856.06 ± 258.19b	0.98
L	6.64 ± 0.12a	0.0035 ± 0.0007b	3600.80 ± 36.77a	3621.92 ± 28.72a	0.98
CK	6.04 ± 0.09b	0.0059 ± 0.0007a	2133.27 ± 155.38c	2138.07 ± 159.11c	0.99

All data are presented as the mean ± SD (standard deviation)

*H* high N application in the sugarcane soil (964 kg ha^−1^), *M* medium N application in the sugarcane soil (482 kg ha^−1^), *L* low N application in the sugarcane soil (96 kg ha^−1^), *CK* no N application in the sugarcane soil (0 kg ha^−1^)

Different letters in the same column indicate significant differences between treatments at *P* < 0.05 among the means of the four treatments

### Bacterial community structure and composition

**Fig. 1:**
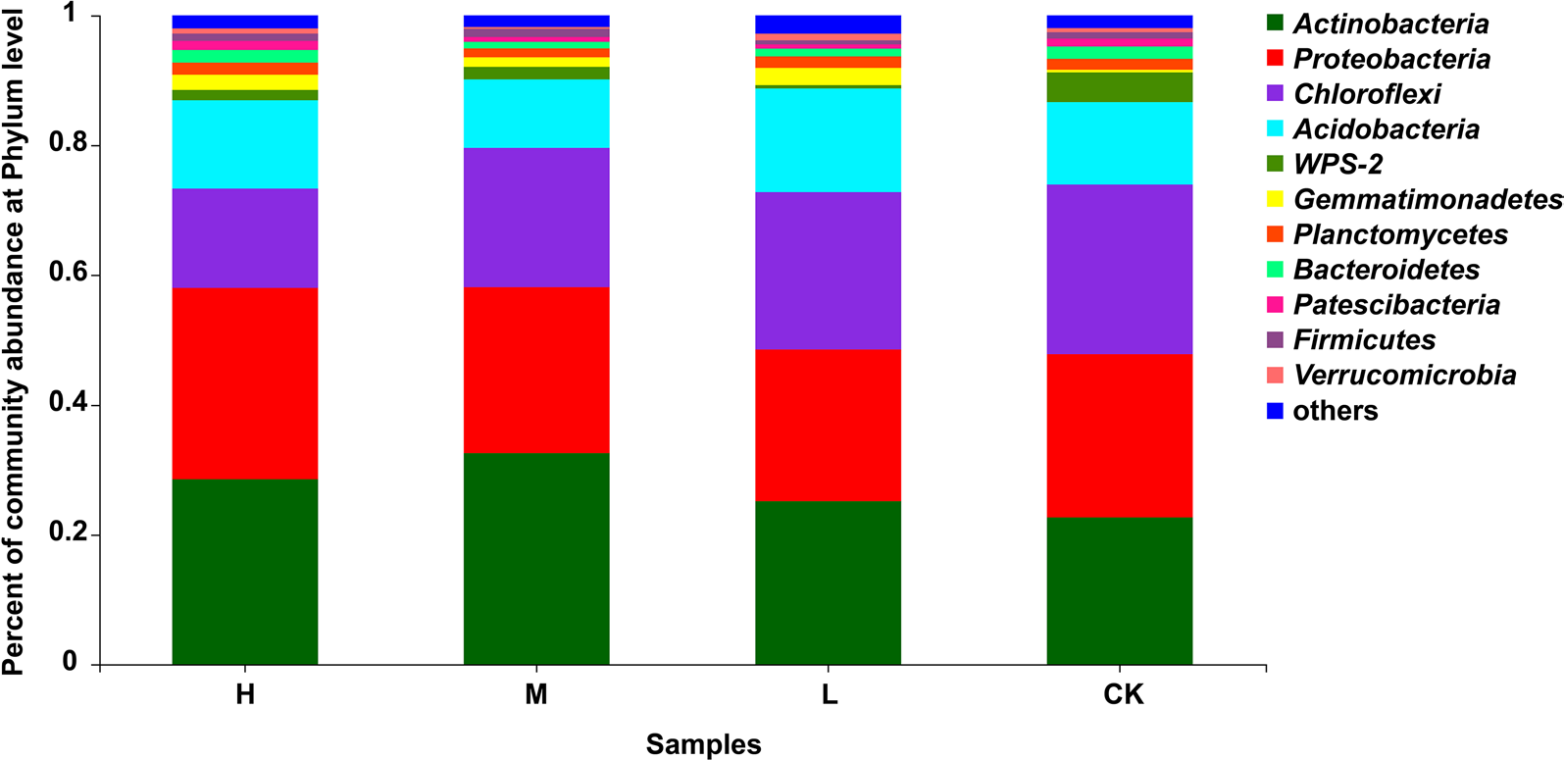
Compositions of soil bacterial communities at phylum level under four N application treatments. H: high N application in the sugarcane soil (964 kg ha^−1^), M: medium N application in the sugarcane soil (482 kg ha^−1^), L: low N application in the sugarcane soil (96 kg ha^−1^), CK: no N application in the sugarcane soil (0 kg ha^−1^)

At the phylum level, soil bacterial communities in the four N applications were dominated (≥ 1%) by *Proteobacteria* (23.3–29.4%), *Actinobacteria* (22.7–32.6%), *Chloroflexi* (15.3–26.2%), *Acidobacteria* (10.6–16.0%), *Gemmatimonadetes* (less than 1–2.67%), *Bacteroidetes* (1.01–1.95%), *Planctomycetes* (1.37–1.87%), *WPS-2* (less than 1–4.58%), *Patescibacteria* (less than 1–1.41%), *Firmicutes* (less than 1–1.25%), *Verrucomicrobia* (less than 1–1.03%) and others (1.79–2.81%) (Fig. 1). Moreover, the numbers of identified bacterial phyla in the H, M, L and CK treatments were 11, 10, 9 and 9, respectively. All these results showed that the N applications not only changed the proportions of dominant soil bacterial phyla, but also altered the compositions of soil bacterial communities. Furthermore, *Proteobacteria*, *Actinobacteria*, *Chloroflexi* and *Acidobacteria* were the four most abundant soil bacterial phyla in sugarcane fields with different N application levels. *Proteobacteria* are easily enriched under high N application conditions, and *Actinobacteria* and *Acidobacteria* sensitively responded to low or medium N applications. By contrast, *Chloroflexi* could be enriched in the soil of sugarcane fields without N application (Additional file 1: Table. S1).

**Fig. 2:**
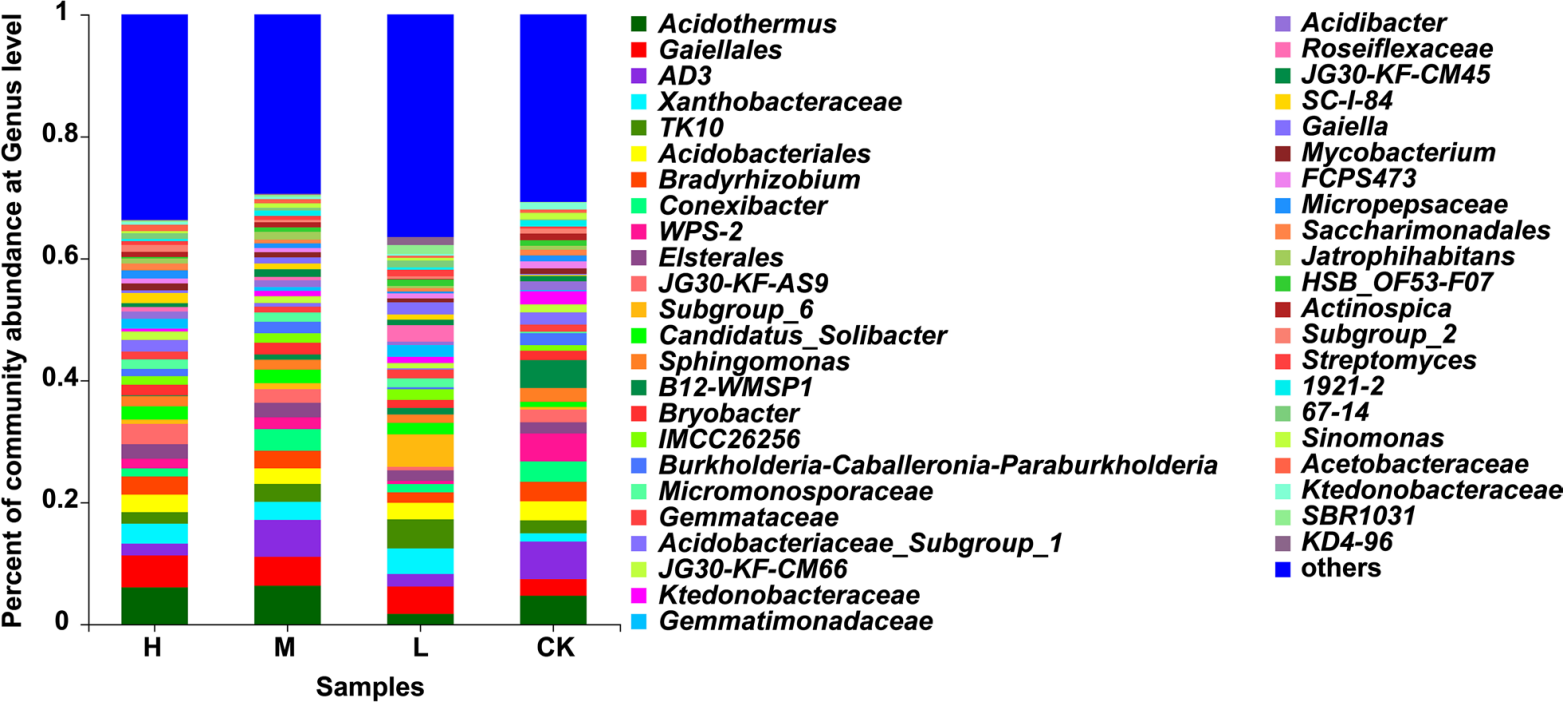
Compositions of soil bacterial communities at genus level under four N application treatments. H: high N application in the sugarcane soil (964 kg ha^−1^), M: medium N application in the sugarcane soil (482 kg ha^−1^), L: low N application in the sugarcane soil (96 kg ha^−1^), CK: no N application in the sugarcane soil (0 kg ha^−1^)

At the genus level, there were 28, 22, 26 and 25 dominant bacterial genera with relative abundances greater than 1% in the high, medium, low and no N application treatments, respectively (Fig. 2). Compared to the CK, the dominant bacterial genera all increased in the high- or low-nitrogen treatments, but they decreased in the medium-nitrogen application treatments. Meanwhile, there were special dominant bacterial genera in every N application treatment, *Mycobacterium*, *SC-I-84*, *Saccharimonadales*, *Micropepsaceae*, *Subgroup_2* and *Acetobacteraceae* were the unique dominant genera in the H treatment. *JG30-KF-CM45* and *Jatrophihabitans* were the unique dominant genera in the M treatment; *Subgroup_6*, *HSB_OF53-F07*, *Streptomyces*, *67–14*, *SBR1031* and *KD4-96* were the unique dominant genera in the L treatment. *FCPS473*, *Actinospica*, *1921–2*, *Sinomonas* and *Ktedonobacteraceae* were the unique dominant genera in the CK treatment. All the above results indicate that the soil bacterial community structure in sugarcane fields could be significantly affected by N input. In particularly, more sensitive effects are triggered by low or high N application (Additional file 1: Table. S2).

**Fig. 3:**
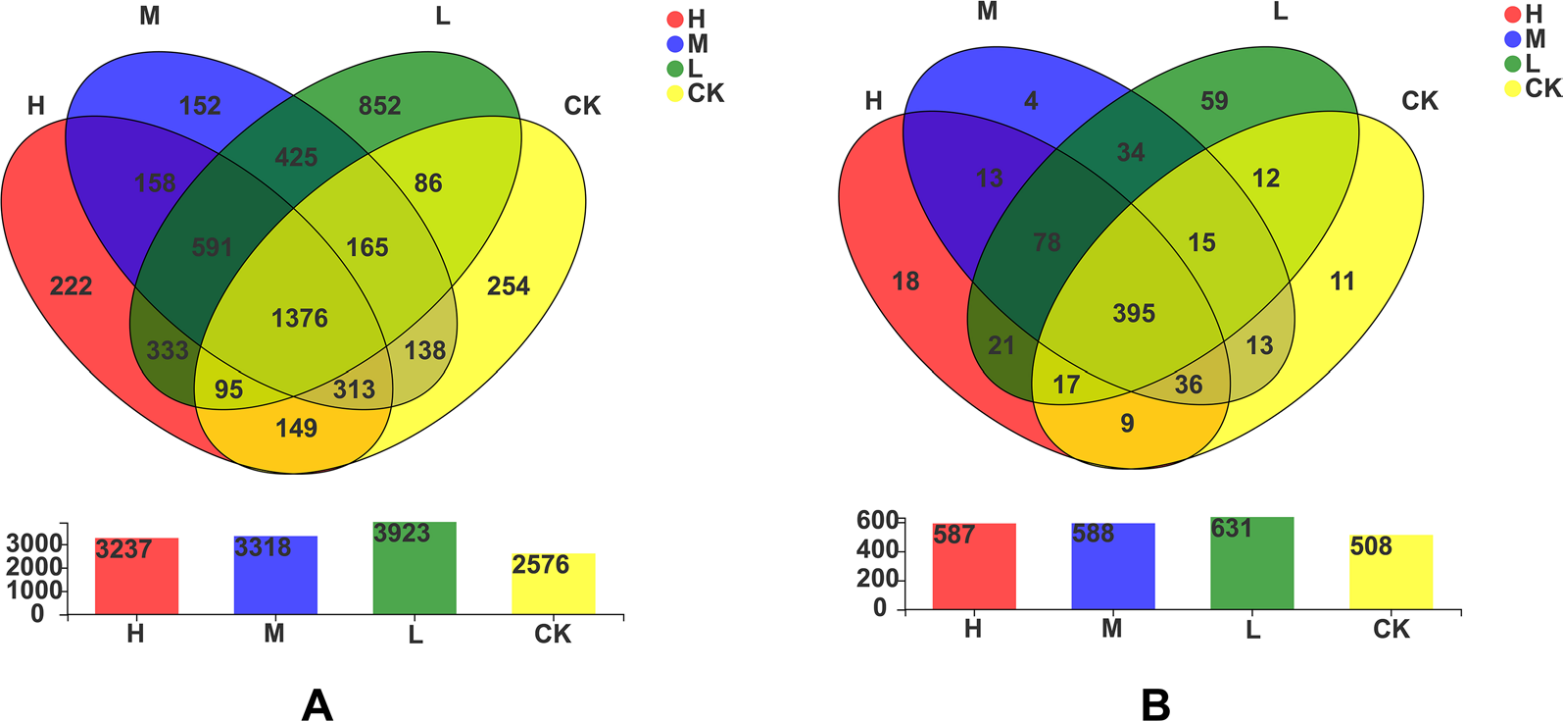
Venn analysis of soil bacteria in sugarcane fields under four N application treatments at the OTU (**A**) and genus (**B**) levels. H: high N application in the sugarcane soil (964 kg ha^−1^), M: medium N application in the sugarcane soil (482 kg ha^−1^), L: low N application in the sugarcane soil (96 kg ha^−1^), CK: no N application in the sugarcane soil (0 kg ha^−1^)

The number of bacteria obtained at the OTU (Operational Taxonomic Units) level under the H, M, L and CK treatments was 3237, 3318, 3923 and 2576, respectively. The numbers of unique bacteria in the H, M, L and CK treatments at the OTU level were 222, 152, 852 and 254, respectively (Fig. 3A). In addition, the numbers of bacteria in the H, M, L and CK treatments at the genus level were 587, 588, 631 and 508, respectively. Moreover, the numbers of unique bacteria in the H, M, L and CK treatments at the genus level were 18, 4, 59 and 11, respectively (Fig. 3B). All the above results suggested that the soil bacterial community structure could be significantly altered by nitrogen application. However, higher nitrogen inputs (964 kg ha^−1^ and 482 kg ha^−1^) were not helpful for improving the number of unique soil bacteria in sugarcane fields. On the contrary, low nitrogen application (96 kg ha^−1^) was more efficient for improving soil bacterial diversity and richness in sugarcane fields.

**Fig. 4:**
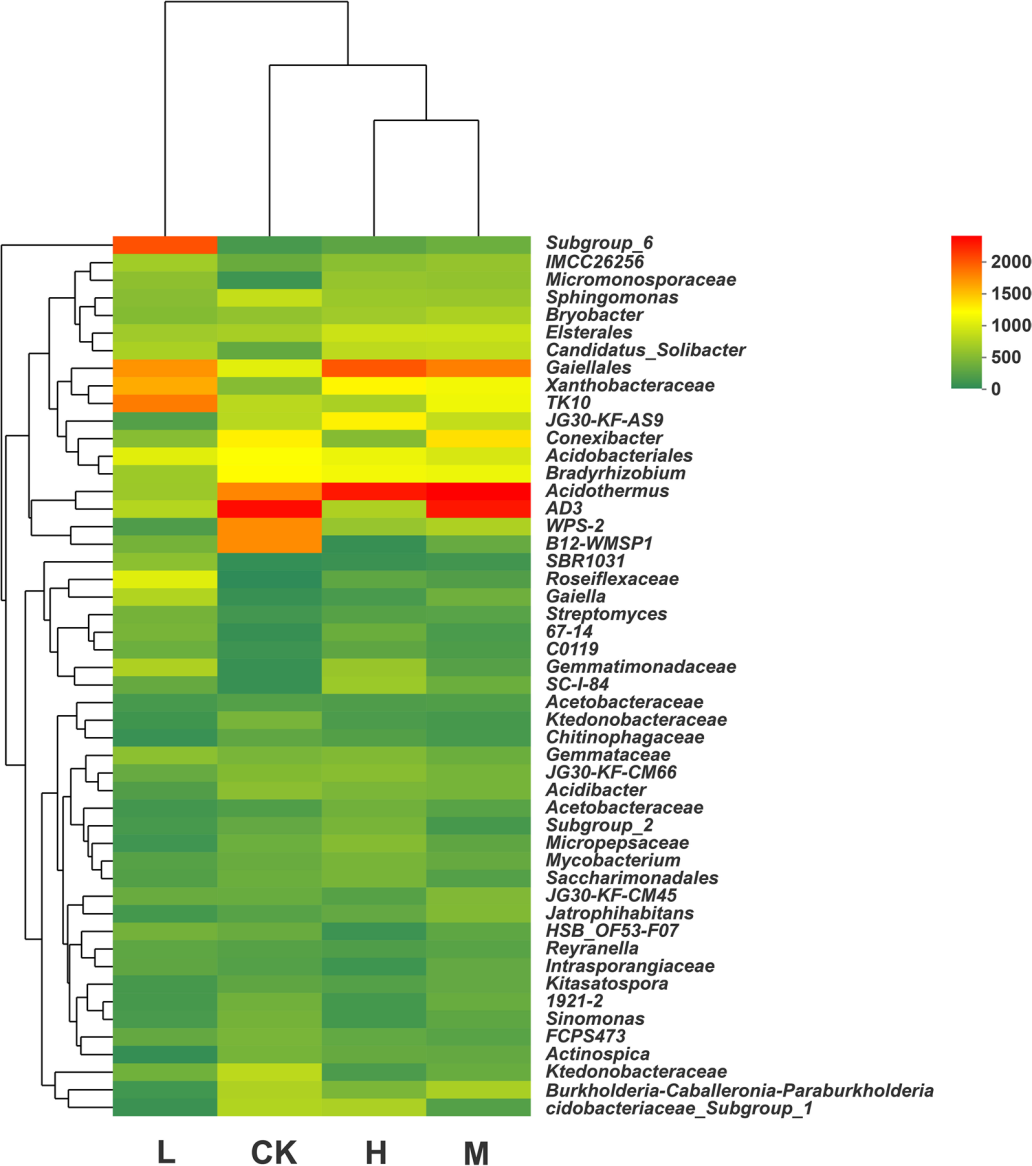
Heatmap of four N application treatments with three replicates based on the relative abundances of the top 50 most abundant genera. A color zone represents relative abundance. H: high N application in the sugarcane soil (964 kg ha^−1^), M: medium N application in the sugarcane soil (482 kg ha^−1^), L: low N application in the sugarcane soil (96 kg ha^−1^), CK: no N application in the sugarcane soil (0 kg ha^−1^)

The top 50 most abundant soil bacteria at the genus level in sugarcane fields under different nitrogen applications were selected to form the heat map (Fig. 4). The horizontal level represents the different treatments, and the longitudinal direction shows the abundance of bacterial species. As seen in Fig. 4, the distribution of soil dominant bacteria under low, high and medium nitrogen applications was different from the distribution of CK, and there was also a difference between each treatment. However, the distribution of soil dominant bacteria was quite similar between the CK and high or medium nitrogen application treatments. In contrast, the composition and abundance of dominant soil bacteria under low nitrogen application changed significantly between CK. This finding indicates that the response of the soil bacterial community structure to nitrogen application is more sensitive under low nitrogen input at 96 kg ha^−1^.

### Principal component analysis

**Fig. 5:**
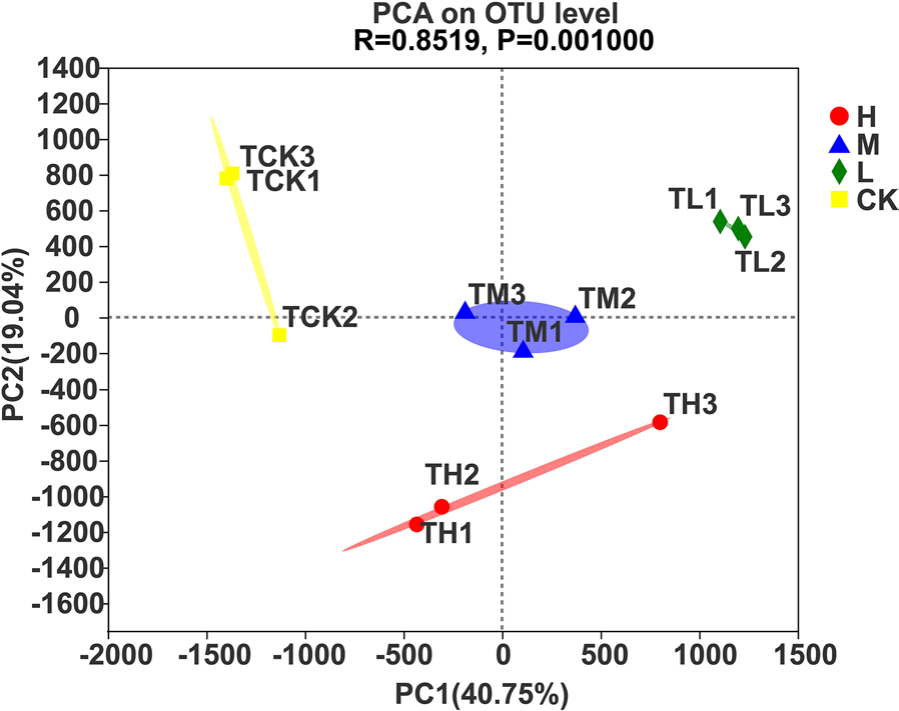
Principal component analysis of the relative abundance of soil bacteria at the OTU level in sugarcane fields under four N application treatments. H: high N application in the sugarcane soil (964 kg ha^−1^), M: medium N application in the sugarcane soil (482 kg ha^−1^), L: low N application in the sugarcane soil (96 kg ha^−1^), CK: no N application in the sugarcane soil (0 kg ha^−1^)

As seen in Fig. 5, the contribution rates of the first and second principal components (PC1 and PC2) were 40.75% and 19.04%, respectively. In addition, low and medium nitrogen applications were distributed mainly in the positive direction of PC1, but high and nonnitrogen applications were found primarily in the negative direction of PC1. Meanwhile, low, medium and nonnitrogen applications were distributed mainly in the positive direction of PC2, and only high nitrogen application was found in the negative direction of PC2. Moreover, only the low nitrogen application treatment was located on the first quadrant, which suggested that low nitrogen application was positively correlated with the first and second principal components.

## Discussion

Guangxi is the main producing area for sugarcane in China, and more than 60% of sugar is produced in Guangxi (Li and Yang 2015). However, overuse of chemical fertilizer and a low utilization rate of fertilizer remain the principal problems in Guangxi sugarcane production (Deng et al. 2017). Our study provides the in situ evidence in searching an eco-friendly method of N application amount for sugarcane production in Guangxi, China.

It is generally accepted that soil enzymes play key biochemical functions throughout the decomposition of organic matter in the soil system (Ellert et al. 1997), not only catalyzing microbial life processes in soil and stabilizing soil structure, decomposing organic waste, forming organic matter and cycling nutrients (Dick et al. 1994) but also maintaining soil ecological physicochemical properties and soil health. However, soil enzyme activities may be affected by land management measures (Carney et al. 2004; Kaye et al. 2005; Acosta-Martínez et al. 2010). The results showed that the activities of *β*-glucosidase and phosphatase in sugarcane field under high N application were all significantly higher than those of CK. However, the activity of aminopeptidase in all N application treatments was significantly lower than that of CK. Furthermore, the activities of *β*-glucosidase and phosphatase in the sugarcane field under medium N application were not significantly different between CKs. However, except for the activities of *β*-glucosidase and aminopeptidase, the activity of acid phosphatase in the sugarcane field under low N stress (L) was significantly higher than that of CK. The findings that the activities of soil enzymes were sensitively affected by N application, but the activities of soil enzymes were not all improved by higher N input. For example, only the activity of acid phosphatase was significantly improved by low N application corroborated the same trends of the previous studies using different nitrogen treatments (Wang et al. 2014; Shi et al. 2016).

Soil microbial biomass is also an important indicator of soil quality to maintain soil fertility and crop productivity (Powlson et al. 1987). The greater the microbial biomass in the soil, the greater is the capacity of the soil to provide nutrients to plants through mineralization of organic nutrients (Dwivedi and Soni 2011). Among these organic nutrients, soil microbial biomass carbon (MBC) can not only promote the formation of new humus with high activity in soil but also reflect the slight change in the soil before the change in soil total carbon content (Doran et al. 1996). Soil microbial biomass N (MBN) can also reflect the availability of soil N and play an important role in the supply and circulation of soil N (Doran et al. 1996). Soil microbial biomass phosphorus (MBP) can reflect the supply level of soil phosphorus (Kwabiah et al. 2003). In addition, although soil microbial biomass phosphorus cannot be directly absorbed and utilized by plants, it can be slowly released as inorganic phosphorus, so it has always been considered the source of available phosphorus in the soil, which is very important for plant growth (Khan and Joergensen 2009). The results showed that soil microbial biomass C and P in the sugarcane field under high N application (H) were significantly decreased, only the microbial biomass N was significantly increased compared with CK. In the medium N application treatment, only the microbial biomass N increased, but the microbial biomass C was significantly decreased, and there was no significant difference in soil microbial biomass P between CK. However, in contrast to CK, the soil microbial biomass C, N and P in the sugarcane field under low N application (L) were all significantly increased. It indicated that low N application (96 kg ha^−1^) was more effective in improving soil fertility than other N applications (964 kg ha^−1^ and 482 kg ha^−1^) in sugarcane fields. Moreover, the finding that medium and high-level N additions although increased soil microbial biomass nitrogen, but medium and high-level N applications decreased soil microbial C and P, which are in agreement with numerous studies in big or small-scales N applications experiments (Yue et al. 2017; Zhou et al. 2017a; Soong et al. 2018).

In addition, microorganisms play a prominent role in agricultural ecosystems, and with the gradual recognition of people, the effect of N fertilizer on soil microorganisms has received increasing attention (Zhou et al., 2017b; Wang et al. 2018). Our results showed that medium (482 kg ha^−1^) and high-level (964 kg ha^−1^) N applications had no significant effect, but low N application (96 kg ha^−1^) showed a significant effect on soil bacterial diversity and richness in this study, which is quite different with numerous studies that have reported the declines in soil bacterial diversity and richness following N enrichment (Ling et al. 2017; Zhang et al. 2018; Wang et al. 2018). The causes of inhibitory N effect on soil bacterial diversity and richness in previous studies attributed to the availability of C decreasing for soil microbes by forming stable compounds (Guo et al. 2017), or decreased soil microbial biomass with excessive N additions (Wang et al. 2018). And our results support that the decreasing of soil microbial biomass C and P is the main reason for nonsignificant effects of medium and higher-level N additions on soil bacterial diversity and richness. Similar as our study, Ramirez et al. (2012) also found that high-level N additions significantly increased the relative abundance of *Actinobacteria* and *Firmicutes*, and N enrichment significantly decreased the relative abundance of *Acidobacteria*, *Verrucomicrobia*, *Cyanobacteria*, and *Planctomycetes*, etc. Moreover, *FCPS473*, *Actinospica*, *1921–2*, *Sinomonas* and *Ktedonobacteraceae* were the unique dominant bacterial genera in CK. In contrast to CK, *_SC-I-84*, *Mycobacterium*, *Micropepsaceae*, *Saccharimonadales*, *Subgroup_2* and *Acetobacteraceae* were the unique dominant soil bacterial genera in the sugarcane field under high-level N application. And *JG30-KF-CM45* and *Jatrophihabitan*; *Subgroup_6*, *HSB_OF53-F07*, *Streptomyces*, *_67-14*, *SBR1031* and *KD4-96* were the unique dominant soil bacterial genera under medium and low N applications, respectively. Meanwhile, the numbers of unique dominant soil bacterial genera or OTUs level in low nitrogen application were all higher than those of CK, medium (482 kg ha^−1^) and high-levels (964 kg ha^−1^) nitrogen application treatments (Fig. 3). As soil bacterial community is sensitive indicators used for the assessment of soil quality (Wu et al. 2017). Our results suggested that soil quality did not decreased in sugarcane field under low N application (96 kg ha^−1^).

Our study focused on the effect of different amount of N application on soil fertility and soil quality in sugarcane fields of Guangxi. We found that soil fertility all could be changed by different N application levels, but the most integral improvement effect only could be found in low N application (96 kg ha^−1^). Moreover, even though soil bacterial richness could be significantly promoted by the medium (482 kg ha^−1^) and high N (964 kg ha^−1^) applications, but soil bacterial diversity could not be significantly improved. On the contrary, soil bacterial diversity and richness all could be improved by low N application (96 kg ha^−1^). In addition, higher abundance of unique soil dominant bacteria could be found low N application (96 kg ha^−1^) which compared to the CK, medium and high-level N applications. These findings suggest that the rate of 96 kg ha^−1^ N application is ecofriendly for sugarcane production in Guangxi.

## Supplementary Information

**Additional file 1: Table S1.** The proportion of soil dominant bacterial communities at the phylum level under four N application treatments (%). **Table S2.** The proportion of soil dominant bacterial communities at genus level under four N application treatments (%).

## Data Availability

The datasets generated during and/or analysed during the current study are available in the NCBI. Raw reads during the current study are deposited in the NCBI Sequence Read Archive (SRA) database (Accession Number: SRP302628).

## Abbreviations

MBC: Soil microbial biomass C
MBN: Soil microbial biomass N
MBP: Soil microbial biomass P
PCA: Principal coordinates analysis
