# Supplementary material for: Response of soil biological properties and bacterial diversity to different levels of nitrogen application in sugarcane fields
Source: AMB Express. 2021 Dec 17;11:172. doi: 10.1186/s13568-021-01331-4 (PMC8683519; doi:10.1186/s13568-021-01331-4)
Supplement: Supplementary file 1 — Additional file 1: Table S1. The proportion of soil dominant bacterial communities at the phylum level under four N application treatments (%). Table S2. The proportion of soil dominant bacterial communities at genus level under four N application treatments (%). [file 13568_2021_1331_MOESM1_ESM.docx]

**Table S1** The proportion of dominant bacterial communities at the phylum level under four N application treatments (%)

| **Phylum** | **H** | **M** | **L** | **CK** |
| --- | --- | --- | --- | --- |
| *Actinobacteria* | 28.5 | 32.6 | 25.2 | 22.7 |
| *Proteobacteria* | 29.4 | 25.5 | 23.3 | 25.1 |
| *Chloroflexi* | 15.3 | 21.5 | 24.3 | 26.2 |
| *Acidobacteria* | 13.6 | 10.6 | 16.0 | 12.7 |
| *WPS-2* | 1.59 | 1.93 | - | 4.58 |
| *Gemmatimonadetes* | 2.35 | 1.48 | 2.67 | - |
| *Planctomycetes* | 1.87 | 1.37 | 1.77 | 1.68 |
| *Bacteroidetes* | 1.95 | 1.01 | 1.20 | 1.91 |
| *Patescibacteria* | 1.41 | - | - | 1.21 |
| *Firmicutes* | 1.11 | 1.25 | - | - |
| *Verrucomicrobia* | - | - | 1.03 | - |
| others | 2.01 | 1.79 | 2.81 | 1.96 |

H: high N application in the sugarcane soil (964 kg ha^-1^), M: moderate N application in the sugarcane soil (482 kg ha^-1^), L: low N application in the sugarcane soil (96 kg ha^-1^), CK: no N application in the sugarcane soil (0 kg ha^-1^).

**Table S2** The proportion of dominant bacterial communities at genus level under four N application treatments (%)

| **Genus** | **H** | **M** | **L** | **CK** |
| --- | --- | --- | --- | --- |
| *Acidothermus* | 6.02 | 6.3 | 1.68 | 4.66 |
| *Gaiellales* | 5.25 | 4.76 | 4.49 | 2.73 |
| *AD3* | 1.94 | 6.03 | 2.05 | 6.16 |
| *Xanthobacteraceae* | 3.27 | 2.97 | 4.20 | 1.36 |
| *TK10* | 1.89 | 2.94 | 4.78 | 2.12 |
| *Acidobacteriales* | 2.88 | 2.56 | 2.72 | 3.13 |
| *Bradyrhizobium* | 2.97 | 2.89 | 1.7 | 3.2 |
| *Conexibacter* | 1.33 | 3.55 | 1.37 | 3.35 |
| *WPS-2* | 1.59 | 1.93 | - | 4.58 |
| *Elsterales* | 2.38 | 2.38 | 1.73 | 1.83 |
| *JG30-KF-AS9* | 3.34 | 2.25 | - | 2.1 |
| *Subgroup_6* | - | - | 5.32 | - |
| *Candidatus_Solibacter* | 2.17 | 2.22 | 1.89 | - |
| *Sphingomonas* | 1.66 | 1.63 | 1.37 | 2.27 |
| *B12-WMSP1* | - | - | 1.08 | 4.58 |
| *Bryobacter* | 1.73 | 1.93 | 1.31 | 1.53 |
| *IMCC26256* | 1.41 | 1.56 | 1.75 | - |
| *Burkholderia-Caballeronia-Paraburkholderia* | 1.18 | 1.87 | - | 1.95 |
| *Micromonosporaceae* | 1.58 | 1.54 | 1.47 | - |
| *Gemmataceae* | 1.28 | - | 1.43 | 1.16 |
| *Acidobacteriaceae_Subgroup_1* | 1.90 | - | - | 2.00 |
| *JG30-KF-CM66* | 1.39 | 1.12 | - | 1.31 |
| *Ktedonobacteraceae* | - | - | 1.02 | 2.16 |
| *Gemmatimonadaceae* | 1.62 | - | 1.93 | - |
| *Acidibacter* | 1.18 | 1.11 | - | - |
| *Roseiflexaceae* | - | - | 2.71 | 1.42 |
| *JG30-KF-CM45* | - | 1.26 | - | - |
| *SC-I-84* | 1.67 | - | - | - |
| *Gaiella* | - | 1.00 | 2.00 | - |
| *Mycobacterium* | 1.14 | - | - | - |
| *FCPS473* | - | - | - | 1.16 |
| *Micropepsaceae* | 1.34 | - | - | - |
| *Saccharimonadales* | 1.13 | - | - | - |
| *Jatrophihabitans* | - | 1.28 | - | - |
| *HSB_OF53-F07* | - | - | 1.06 | - |
| *Actinospica* | - | - | - | 1.10 |
| *Subgroup_2* | 1.13 | - | - | - |
| *Streptomyces* | - | - | 1.08 | - |
| *1921-2* | - | - | - | 1.04 |
| *67-14* | - | - | 1.13 | - |
| *Sinomonas* | - | - | - | 1.09 |
| *Acetobacteraceae* | 1.04 | - | - | - |
| *Ktedonobacteraceae* | - | - | - | 1.12 |
| *SBR1031* | - | - | 1.45 | - |
| *KD4-96* | - | - | 1.32 | - |
| others | 33.69 | 29.41 | 36.51 | 30.75 |

H: high N application in the sugarcane soil (964 kg ha^-1^), M: moderate N application in the sugarcane soil (482 kg ha^-1^), L: low N application in the sugarcane soil (96 kg ha^-1^), CK: no N application in the sugarcane soil (0 kg ha^-1^).
